# Isolation of Novel Trypanosomatid, *Zelonia australiensis* sp. nov. (Kinetoplastida: Trypanosomatidae) Provides Support for a Gondwanan Origin of Dixenous Parasitism in the Leishmaniinae

**DOI:** 10.1371/journal.pntd.0005215

**Published:** 2017-01-12

**Authors:** Joel Barratt, Alexa Kaufer, Bryce Peters, Douglas Craig, Andrea Lawrence, Tamalee Roberts, Rogan Lee, Gary McAuliffe, Damien Stark, John Ellis

**Affiliations:** 1 School of Life Sciences, University of Technology Sydney, Sydney, New South Wales, Australia; 2 Insect Research Facility, University of Technology Sydney, Sydney, New South Wales, Australia; 3 Department of Biological Sciences, University of Alberta, Edmonton, Alberta, Canada; 4 Faculty of Veterinary Science, University of Sydney, Sydney, New South Wales, Australia; 5 Department of Medical Entomology, University of Sydney & Pathology West - ICPMR, Westmead Hospital, Westmead, New South Wales, Australia; 6 St. Vincent's Hospital Sydney, Division of Microbiology, Sydney, New South Wales, Australia; 7 Centre for Infectious Diseases and Microbiology Laboratory Services, ICPMR, Westmead Hospital, Westmead, New South Wales, Australia; 8 Microbiology Department, Royal Darwin Hospital, Darwin, Northern Territory, Australia; Charité University Medicine Berlin, GERMANY

## Abstract

The genus *Leishmania* includes approximately 53 species, 20 of which cause human leishmaniais; a significant albeit neglected tropical disease. Leishmaniasis has afflicted humans for millennia, but how ancient is *Leishmania* and where did it arise? These questions have been hotly debated for decades and several theories have been proposed. One theory suggests *Leishmania* originated in the Palearctic, and dispersed to the New World via the Bering land bridge. Others propose that *Leishmania* evolved in the Neotropics. The Multiple Origins theory suggests that separation of certain Old World and New World species occurred due to the opening of the Atlantic Ocean. Some suggest that the ancestor of the dixenous genera *Leishmania*, *Endotrypanum* and *Porcisia* evolved on Gondwana between 90 and 140 million years ago. In the present study a detailed molecular and morphological characterisation was performed on a novel Australian trypanosomatid following its isolation in Australia’s tropics from the native black fly, *Simulium* (*Morops*) *dycei* Colbo, 1976. Phylogenetic analyses were conducted and confirmed this parasite as a sibling to *Zelonia costaricensis*, a close relative of *Leishmania* previously isolated from a reduviid bug in Costa Rica. Consequently, this parasite was assigned the name *Zelonia australiensis* sp. nov. Assuming *Z*. *costaricensis* and *Z*. *australiensis* diverged when Australia and South America became completely separated, their divergence occurred between 36 and 41 million years ago at least. Using this vicariance event as a calibration point for a phylogenetic time tree, the common ancestor of the dixenous genera *Leishmania*, *Endotrypanum* and *Porcisia* appeared in Gondwana approximately 91 million years ago. Ultimately, this study contributes to our understanding of trypanosomatid diversity, and of *Leishmania* origins by providing support for a Gondwanan origin of dixenous parasitism in the Leishmaniinae.

## Author Summary

The genus *Leishmania* includes approximately 53 species, 20 of which cause human leishmaniais, a significant disease that has afflicted humans for millennia. But how ancient is *Leishmania* and where did it arise? Some suggest *Leishmania* originated in the Palearctic. Others suggest it appeared in the Neotropics. The Multiple Origins theory proposes that separation of certain Old World and Neotropical species occurred following the opening of the Atlantic. Others suggest that an ancestor to the Euleishmania and Paraleishmania appeared on Gondwana 90 to 140 million years ago (MYA). We performed a detailed molecular and morphological characterisation of a novel Australian trypanosomatid. This parasite is a sibling to the Neotropical *Zelonia costaricensis*, a close relative of *Leishmania*, and designated as *Zelonia australiensis* sp. nov. Assuming *Z*. *costaricensis* and *Z*. *australiensis* split when Australia and South America separated, their divergence occurred between 36 and 41 MYA. Using this event as a calibration point for a phylogenetic time tree, an ancestor of the dixenous Leishmaniinae appeared in Gondwana ~ 91 MYA. This study contributes to our understanding of trypanosomatid diversity by describing a unique Australian trypanosomatid and to our understanding of *Leishmania* evolution by inferring a Gondwanan origin for dixenous parasitism in the Leishmaniinae.

## Introduction

The success of *Leishmania* species, the complexity of their dixenous life cycle, and the intricacy of their host-parasite interactions implies a relationship between host, parasite and vector that has evolved over millions of years, certainly predating the appearance of humankind. Evidence for this ancient origin was first identified in the form of *Paleoleishmania proterus*; a trypanosomatid discovered in a fossilised *Palaeomyia burmitis* sand fly that became trapped in Burmese amber approximately 100 million years ago (MYA) [1]. A second fossilised specimen of the extinct sand fly *Lutzomyia adiketis* contained a trypanosomatid parasite assigned the name *Paleoleishmania neotropicum* [2]. This specimen was preserved in amber from the Dominican Republic and was dated at 20 to 30 million years old [2]. While these findings provide insights into the ancient origins of *Leishmania*, the evolutionary and biogeographical history of this genus remains a hotly debated topic, and multiple theories have been proposed [3–6].

The Palaearctic origins theory suggests that *Leishmania* originated in the Old World and dispersed to the New World via the Bering land bridge which was open during the Eocene epoch [6–8]. Amastigotes of the ~100 million year old *Paleoleishmania proterus* were observed in Cretaceous reptilian blood [9], supporting that the reptile-infecting *Sauroleishmania* subgenus evolved first in the Palearctic. However, this requires that the *Sauroleishmania* form a sister clade to all other *Leishmania* species [3, 7], and implies that adaptation to mammals, possibly murid rodents, occurred later when reptiles declined during the global cooling episode that denotes the Eocene to Oligocene transition [6, 8, 10]. Alternatively, the Neotropical origins hypothesis suggests *Leishmania* appeared in the Neotropics between 34 and 46 MYA and was dispersed to the Nearctic by rodents (i.e. porcupines) via the Panamanian land bridge [11]. The parasites were then dispersed further, from the Nearctic to the Palaearctic via the Bering land bridge [3, 6].

The Multiple Origins hypothesis, also known as the Neotropical/African Origins hypothesis [6], considers the origins of the Euleishmania, comprising the *Leishmania*, *Viannia*, and *Sauroleishmania* subgenera, and the Paraleishmania [7] which presently includes *Endotrypanum* and the newly established genus, *Porcisia* Shaw, Camargo and Teixeira, 2016 [12]. This hypothesis supposes that the Euleishmania and Paraleishmania existed as separate lineages prior to the breakup of Gondwana. Upon the opening of the Atlantic Ocean, the Euleishmania evolved into the *Sauroleishmania* and *Leishmania* subgenera in the Old World, and the *Viannia* subgenus evolved from the Euleishmania that remained in the New World [7]. This theory also supposes that an ancestor of the few known Neotropical *Leishmania* (*Leishmania*) species was later dispersed from the Old World to the New World via the Bering land bridge [3, 6]. The Supercontinents hypothesis represents a variation of the Multiple Origins theory, and proposes that the Euleishmania and Paraleishmania diverged approximately 90 to 100 MYA, and that an ancestor to *Leishmania*, *Endotrypanum* and *Porcisia* evolved from a monoxenous trypanosomatid on Gondwana between 90 and 140 MYA [3]. This hypothesis was discussed several years ago by Yurchenko *et al*. [4], though more recently explored by Harkins *et al*. [3], who also provided phylogenetic support. Inclusion of an Australian *Leishmania* species in phylogenies from that study also allowed calibration of time trees at a speciation event (a node) that likely arose when Australia became completely separated from South America, via Antarctica, approximately 40 MYA [3]. However, the separation of these continents was a highly protracted event, beginning during the early Cretaceous period and resulting in a large rift valley between Australia and Antarctica as early as 125 to 105 MYA [13]. Consequently, calibration of this node at 40 MYA represents a minimum time point for the vicariant event that separated the Australian *Leishmania* parasite from its ancestors in the Neotropics.

There has been an intense effort amongst trypanosomatid taxonomists in recent years to increase our knowledge of trypanosomatid diversity and better understand the evolutionary relationships between members of this important group of parasites [12, 14, 15]. These endeavours have required detailed molecular and morphological characterisation of newly isolated species to avoid misclassification and subsequent confusion for later investigators [15]. This work has led to several new developments, including establishment of new genera and the reassignment of "old" parasites to different genera [12, 14–19]. Despite these recent advances, knowledge of Australia's indigenous Leishmaniinae remains incredibly scarce. Extended periods of geographical isolation have resulted in Australia's unique and often peculiar fauna. Indeed, this uniqueness is reflected in Australia's native *Leishmania* parasite which, curiously, is thought to be transmitted in the bite of a day feeding midge (Diptera: Ceratopogonidae), rather than a phlebotamine sand fly [20]. Given Australia's unique fauna, surveying its insects for endogenous trypanosomatids could contribute markedly to our understanding of trypanosomatid diversity and uncover evolutionary relationships that were previously elusive.

As a contribution to these efforts, we describe the detailed molecular and morphological characterisation of a novel trypanosomatid isolated from the Australian native black fly, *Simulium* (*Morops*) *dycei* Colbo, 1976. Phylogenetic analyses confirmed this parasite as a sibling species to *Leptomonas costaricensis*; a trypanosomatid previously isolated from a reduviid bug in Costa Rica [4]. In a recent appraisal of trypanosomatid taxonomy, Espinosa *et al*. [12] argued that *L*. *costaricensis* was phylogenetically distant from other *Leptomonas* spp. and should be placed in a separate genus. Consequently, the genus *Zelonia* n. gen Shaw, Camargo and Teixeira (2016) [12] was established to accommodate this organism (henceforth *Zelonia costaricensis*) and its nearest relatives. Accordingly, the Australian parasite isolated in this study was assigned the name *Zelonia australiensis* sp. nov. Assuming that the separation of *Z*. *costaricensis* and *Z*. *australiensis* occurred as a result of vicariance, when Australia and South America separated, we suggest their divergence took place between 36 and 41 MYA, at least [21]. Using this event as the calibration point for a phylogenetic time tree, the clade containing the dixenous parasites *Leishmania*, *Endotrypanum* and *Porcisia* i.e. the Euleishmania and Paraleishmania, was estimated to have diverged from a monoxenous ancestor in Gondwana during the mid-Cretaceous, approximately 91 MYA. Ultimately, this study contributes to our understanding of trypanosomatid diversity, and of *Leishmania* origins, by providing support for a Gondwanan origin of dixenous parasitism in the Leishmaniinae.

## Materials and Methods

### Study location and insect trapping

Insect collection was performed following approval by the University Technology Sydney Animal Care and Ethics Committee. Insect trapping was performed near the location selected by Dougall *et al*. [20] (Table 1, S1 File) as it was considered suitable for the isolation of other tropical trypanosomatids and would provide an opportunity to re-isolate the Australian *Leishmania* parasite [22], thereby confirming its persistence in the region. Note that at the time of writing, the name *Leishmania* ‘australiensis’ had been used to describe this Australian *Leishmania* parasite in the scientific literature [6], and in an Australian government document [23], in the absence of any formal description. Consequently, the name *Leishmania* ‘australiensis’ is a *nomen nudum* and is no longer available as a species name. To prevent continued use of this *nomen nudum*, the present study includes a formal description of this Australian *Leishmania* species, referred to henceforth as *Leishmania macropodum* sp. nov., Barratt, Kaufer & Ellis 2017.

**Table 1:** Precise coordinates of insect trap sites and trapping times.

Trap site #	Latitude	Longitude	Elevation	Trapping times
1	-12°42’29.6100”	130°59’37.8240”	26.18 m	9.45 am– 11.30 am
				11.30 am– 2.00 pm
2	-12°42’26.7186”	130°59’38.3382”	21.24 m	10.00 am– 11.40 am
				11.40 am– 2.15 pm
3	-12°42’30.9960”	130°59’46.5534”	21.16 m	10.30 am– 12.00 pm
				12.00 pm– 2.30 pm

### Insect identification

Trapped midges and flies were identified with the aid of keys and descriptions [20, 24–27]. Fly specimens were dissected and mounted using the method described by Craig *et al*. [28]. In some cases, DNA was extracted from flies for barcoding purposes prior to identification by morphology. A DNA extraction method described by Lawrence *et al*. [29] (S1 File) was employed that conserved the exoskeleton for downstream morphological identification.

### Cultivation of parasites from insects

Insects were pooled and crushed with a spatula in ~200 μL of PBS. The resulting suspension was used to inoculate a *Leishmania* culture medium based on the medium previously described by Dougall *et al*. [20]. The parasite cultures obtained were initially contaminated with a *Fusarium* sp. fungus. As the parasite cells outnumbered the fungi, the cultures were axenised by serial dilution such that the fungi were diluted out resulting in a pure promastigote culture. To facilitate downstream promastigote counting experiments, a liquid medium was developed and optimised to establish the ideal haemoglobin content (S1 File).

### Light microscopy and transmission electron microscopy

To examine the morphology of cultured promastigotes, a Leishman stain was performed (Sigma-Aldrich) on cell-dense promastigote cultures, in accordance with the manufacturer’s instructions. Cell morphology was examined by oil emersion light microscopy (1000X magnification) using a Leica DM1000 microscope (Leica Microsystems). To examine their ultrastructural features, cultured promastigotes were embedded in low melting point agarose and prepared for transmission electron microscopy using standard procedures (S1 File). Following this, ultrathin sections were cut from the agarose and examined using a Hitachi H-7650 Transmission Electron Microscope (USA).

### DNA extraction and Polymerase Chain Reaction (PCR)

For extraction of total DNA from parasites, approximately 1 mL of dense promastigote culture was placed in a 1.5 mL tube and the cells were pelleted by centrifugation at 300 g for 15 minutes. The supernatant was discarded and DNA was extracted from the pellet using an EZ1 DNA tissue extraction kit (QIAGEN) and a BioRobot EZ1 DNA extracting robot (QIAGEN) according to the manufacturer’s instructions. The DNA was eluted in a volume of 50 μL for downstream PCR analysis. PCR primers were designed to amplify the *18S rRNA* gene and three protein coding genes; the glycosomal glyceraldehyde 3-phosphate dehydrogenase (*gGAPDH*), RNA polymerase II largest subunit (*RPOIILS*), and heat shock protein 70 (*HSP70*) genes (Table 2). To generate PCR products from insects for barcoding purposes, a set of previously published primers were used to amplify fragments of the cytochrome C oxidase subunit I (*COI*) and II (*COII*) genes, the *18S rRNA* gene, and the *28S rRNA* gene (Table 2). Each PCR was prepared using reagents provided in the BIOTAQ PCR Kit (Bioline) (S1 File). The PCR products were subjected to electrophoresis on 2% agarose gels stained with GelRed, and visualised under UV light.

**Table 2:** PCR primers used in this study.

Target	Primer name	Primer sequence (5’ to 3’)	Annealing Temp.	Amplicon size	Reference
**Parasite**					
*gGAPDH*	LeptoC-1	ATCGTGATGGGCGTGAAC	57°C	~450	This study
	LeptoC-2	TGCCCTTCATGTACGTCT			
*RPOIILS*	RPOIILS-1	AACAAGCTCAAGATGAACCTG	57°C	~545	This study
	RPOIILS-2	CATTGCGCTGGTTCTTGCT			
*18S rRNA*	SSU-1	ATCTGCGCATGGCTCATTAC	57°C	~1155	This study
	SSU-2	CACACTTTGGTTCTTGATTGA			
*HSP70*	Hsp70-1	ACGCTGCTGACGATCGAC	59°C	~850	This study
	Hsp70-2	ACACGTTCAGGATGCCGTT			
ITS1 DNA	LITSR	CTGGATCATTTTCCGATG	58°C	~300	[32]
	L5.8S	TGATACCACTTATCGCACTT			
**Fly**					
*COX I*	LCO1490	GGTCAACAAATCATAAAGATATTGG	52°C	~700	[103]
	HCO2198	TAAACTTCAGGGTGACCAAAAAATCA			
*COX II*	TL2-J-3034	ATTATGGCAGATTAGTGCA	54°C	~810	[104]
	TK-N-3785	GTTTAAGAGACCAGTACTTG			
*18S rRNA*	B18S_F	TTTTATGCAAGCCAAGCACA	63°C	~920	[104]
	B18S_R	TGGGAATTCCAGGTTCATGT			
*28S rRNA*	B28S_F	GAAAAGGGAAAAGTCCAGCAC	63°C	~890	[104]
	B28S_R	CACATTTTATGCGCTCATGG			
**Plasmid sequencing primers**					
Cloning vector	T3	ATTAACCCTCACTAAAGGGA	N/A	N/A	N/A
	T7	TAATACGACTCACTATAGGG			

### Sequencing of PCR products

The PCR products were excised from agarose gels using a sterile scalpel blade. Amplicons were extracted from gel slices using a QIAquick Gel Extraction Kit (QIAGEN) according to the manufacturer’s instructions. Sequencing was performed by the service provider Macrogen (South Korea) on an ABI 3730XL capillary sequencer. Ambiguous, low quality bases were manually trimmed from the ends of sequences which were then assembled using CAP3 [30]. Sequences generated from PCR amplicons of *gGAPDH* and *RPOIIL* displayed several ‘dual-peaks’, where two bases were superimposed at the same base position along the sequence. Furthermore, the multi-copy ITS1 DNA sequences of trypanosomatids can differ between copies, making direct sequencing of ITS1 amplicons difficult [31]. Cloning of these amplicons was performed to overcome this issue, so that individual clones could be sequenced. These amplicons were cloned using a TOPO TA cloning kit for sequencing (Thermo Fisher Scientific). Cloning reactions were prepared according to the manufacturer’s instructions (S1 File), and sequencing of cloned PCR fragments was carried out directly from the purified plasmid, twice in the forward and reverse directions, by the service provider Macrogen. Sequencing was performed using the universal T3 and T7 primers (Table 2), which possess priming sites flanking the amplicon insertion site.

### Restriction Fragment Length Polymorphism (RFLP) analysis

A PCR-RFLP assay targeting the Leishmaniinae ITS1 DNA, previously described by Schönian *et al*. [32] was employed to further characterise the newly isolated trypanosomatid (S1 File). As controls for comparison, this assay was carried out on genomic DNA from *Leptomonas seymouri*, *Leishmania turanica*, *Leishmania major* and *Wallacemonas collosoma* (previously *Leptomonas collosoma*). These DNA specimens were kindly provided by Professor Larry Simpson (University of California, Los Angeles) and date back to the study by Lake *et al*. [33]. *Leishmania donovani* DNA provided by the Department of Microbiology at St Vincent’s Hospital, Sydney was also included for comparison. The restriction fragments were subjected to agarose gel electrophoresis on a 3% gel stained with GelRed and visualised under UV light.

### Phylogenetic analysis

Phylogenetic trees were constructed to infer the evolutionary relationship between this newly isolated trypanosomatid and other related parasites. S1 Table lists all GenBank accession numbers for sequences generated in this study and those published by others that were used to construct phylogenetic trees. Multiple sequence alignments were performed using the MEGA software package, version 7.0.14 [34]. Alignments were manually curated to improve accuracy, and phylogenetic analysis was performed using MEGA. Trees were inferred using three methods: the Maximum Likelihood (ML) method based on the Tamura-Nei model [35], the Minimum Evolution (ME) method [36], and the Neighbour-Joining (NJ) method [37]. For ML trees, initial trees for the heuristic search were obtained automatically by applying the Neighbor-Join and BioNJ algorithms to a matrix of pairwise distances estimated using the Maximum Composite Likelihood (MCL) approach, and then selecting the structure with superior log likelihood values. For ME trees, the evolutionary distances were computed using the MCL method [38], and were searched using the Close-Neighbor-Interchange algorithm at a search level of 2 [39]. The Neighbor-Joining algorithm was used to generate the initial ME tree [37]. For NJ trees, the evolutionary distances were also computed using the MCL method [38]. Time trees were generated using the RelTime method [40].

## Results

### Insect identification, fly molecular analysis and parasite isolation

**Fig 1:**
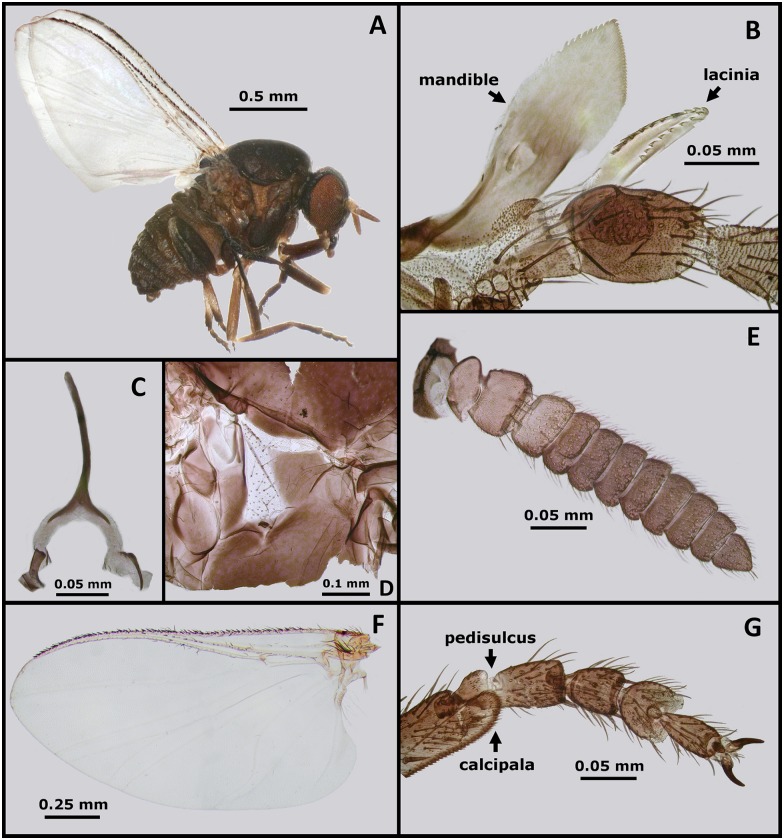
Morphology of a female *Simulium* (*Morops*) *dycei*, Colbo 1976. (A) Habitus of *S*. (*M*.) *dycei* female. (B) Mandible and lacinia of *S*. (*M*.) *dycei* female. (C) Genital fork of *S*. (*M*.) *dycei* female. (D) Anepisternal (pleural) membrane of *S*. (*M*.) *dycei* female. (E) Antenna of *S*. (*M*.) *dycei* female. (F) Wing of *S*. (*M*.) *dycei* female. (G) Hind leg tarsomeres of *S*. (*M*.) *dycei* female showing the pedisulcus and calcipala.

Seventy-nine *Forcipomyia* (*L*.) spp. midges were collected from traps though none were recovered directly from the fur of macropods. Fifty *Forcipomyia* (*L*.) spp. were pooled in three groups (of 10, 20 and 20) for parasite culture, though all were negative for promastigotes after 2 weeks incubation. Other species recovered in traps included *Culicoides* spp., *S*. (*M*.) *dycei* (Fig 1), mosquitoes, phlebotomine sand flies and several others. *Simulium* (*M*.) *dycei* were particularly common, with over 120 specimens recovered from traps and 20 aspirated directly from the fur of macropods. Simuliidae are known vectors of other important parasites [41], and are common pests [42]. Consequently, the observation of *S*. (*M*.) *dycei* commonly biting macropods around the eyes, ears, wrists and feet also encouraged its selection for further study. PCR products were sequenced from the *COI*, *COII*, *18S rRNA*, and *28S rRNA* genes of two female *S*. (*M*.) *dycei* specimens (Fly A and Fly B) (GenBank Accessions KY288010 to KY288017). The identity of these GenBank depositions as belonging to *S*. (*M*.) *dycei* was confirmed beyond a doubt by morphological examination of the exoskeletons following DNA extraction (S1 Fig). Three cultures were prepared from *S*. (*M*.) *dycei* (pools of 20 flies), and one culture was positive for *Leishmania*-like promastigotes after 2 weeks incubation. All remaining specimens of *S*. (*M*.) *dycei* (n = 24) were tested for Leishmaniinae DNA using the PCR assay described by Schönian *et al*. [32], though all returned a negative result.

### Effect of haemoglobin on growth

**Fig 2:**
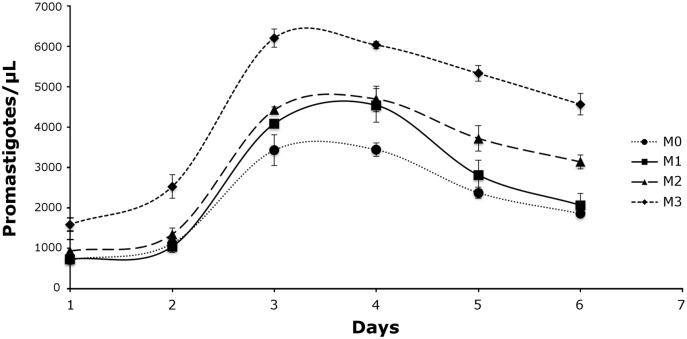
Effect of haemoglobin on promastigote growth. Promastigotes were cultured in triplicate in three media differing in haemoglobin content; M1 (0.0099 g/L), M2 (0.495 g/L) and M3 (0.99 g/L). These media were accompanied by a negative control medium containing no haemoglobin (M0). Promastigote growth seems related to haemoglobin concentration, with the most rigorous growth and highest cell densities observed in M3; the media with the highest haemoglobin concentration. The slowest growth and lowest cell densities were observed in M0, the negative control.

Promastigote growth was investigated in four liquid media differing in haemoglobin content (M0 to M3) (S1 File). Growth was observed in all media including M0 which contained no haemoglobin although the highest cell densities were observed in M3, which contained the highest haemoglobin concentration (Fig 2). In all media, promastigote growth peaked at day 3 and numbers plateaued by day 4. Promastigote numbers steadily decreased until the experiment was terminated on day 6.

### Promastigote morphology

**Fig 3:**
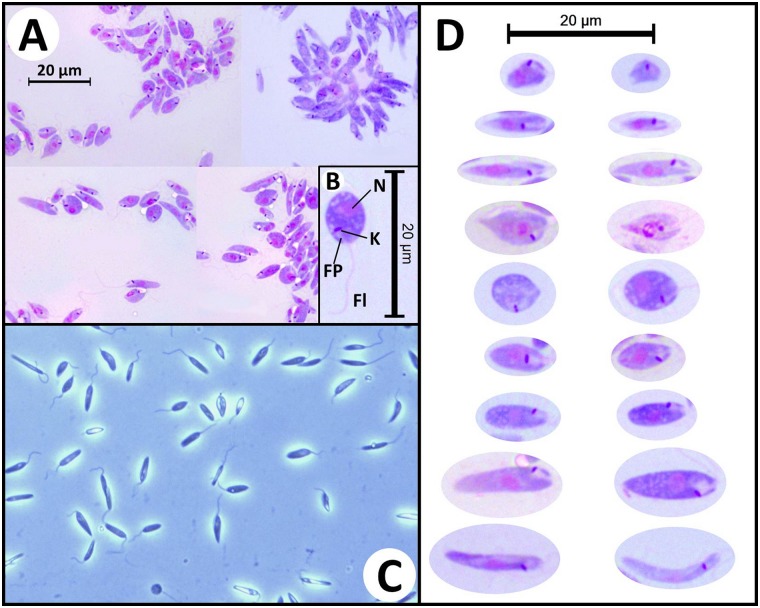
Morphology of trypanosomatid cells in axenic cultures. (A) Photomicrographs of Leishman stained *Zelonia australiensis* promastigotes cultured in M3, viewed under oil emersion microscopy (1000X magnification). (B) Photomicrograph of a round promastigote with gross morphological characteristics indicated including the nulcleus (N), kinetoplast (K), flagellar pocket (FP), and flagellum (Fl). (C) Wet mount photomicrograph of live axenically cultured *Zelonia australiensis* promastigotes viewed under phase contrast microscopy (400X magnification) showing several forms. (D) Photomicrographs of the various *Z*. *australiensis* forms as seen in Leishman stained slides, prepared from axenically cultured parasites. The parasite shows a high degree of pleomorphism in culture. This has been reported for other trypanosomatids, and limits the use of morphology for classification of these organisms [16, 101].

**Fig 4:**
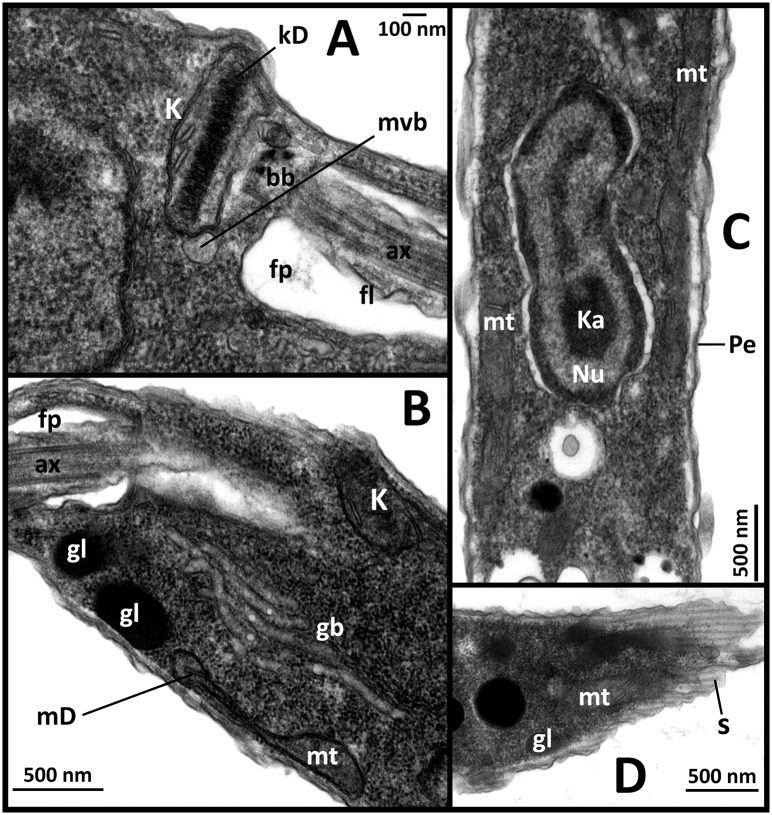
Transmission electron micrographs of promastigotes showing fine detail. (A) Fine structure closely associated with the flagellum (fl) including the kinetoplast (K), basal body (bb), flagella pocket (fp), axonemes (ax), kinetoplast disk (kD) and a multivesicular body (mvb). (B) Fine cell structures including the golgi body (gb), glycosomes (gl) and mitochondria (mt). Mitochondrial DNA (mD) is visible within the mitochondria and kinetoplast (K). (C) Longitudinal cross-section of promastigote showing the nucleus (Nu), elongated mitochondria (mt), karyosome (Ka) and pellicle (Pe). (D) Example of striated pattern cause by sectioning of promastigote across the subpellicular microtubules (s).

Leishman stained smears and wet preparations of cultured parasites revealed several cell morphotypes. Images of these forms are provided in Fig 3. Transmission electron microscopy performed on cultured promastigotes confirmed the presence of ultrastructural features consistent with the Leishmaniinae and similar to the descriptions of *Zelonia costaricensis* (Fig 4) [4].

### Molecular characterisation of parasites

**Fig 5:**
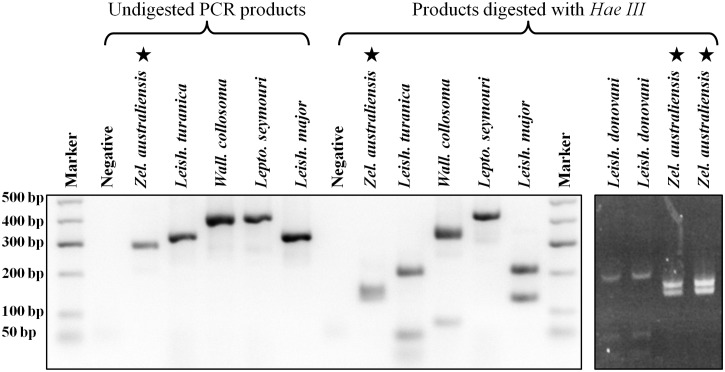
PCR-RFLP analysis of the newly isolated parasite and other Leishmaniinae. Comparison of PCR products and *Hae III* restriction fragments generated for several Leishmaniinae, including *Leptomonas seymouri* and *Wallacemonas collosoma*. Stars indicate the PCR products and restriction fragments generated for *Zelonia australiensis*. Samples were run against a 50 bp Hyperladder molecular weight marker (Bioline). An additional gel image (far right) includes the *Hae III* digested PCR product from *Z*. *australiensis* compared to that of *Leishmania donovani*.

BLAST searches carried out on the parasite sequences generated in this study (GenBank Accessions KY273490 to KY273515) suggested the parasite was of the subfamily Leishmaniinae. The PCR-RFLP assay generated a restriction pattern for the isolate that differed when compared to that produced for the other species tested (Fig 5). Seventeen unique ITS1 DNA clones (GenBank Accessions KY273499 to KY273515), four unique *gGAPDH* clones (GenBank Accessions KY273493 to KY273496) and three unique *RPOIILS* clones (GenBank Accessions KY273490 to KY273492), were generated. The *L*. *seymouri* sequences generated in this study for *gGAPDH*, *HSP70* and the *18S rRNA* genes (GenBank Accessions KY273516, KY273519 and KY273517, respectively) were identical to *Leptomonas* spp. sequences already available in GenBank (Accessions: AF047495, FJ226475 and KP717895, respectively), supporting the accuracy of sequences generated using this workflow. However, the *RPOIILS* sequence generated in this study (GenBank Accession: KY273518) differed by six bases to a previously published *L*. *seymouri* sequence which may indicate the sequence was derived from a different strain (GenBank Accession: AF338253).

### Phylogenetic analysis

**Fig 6:**
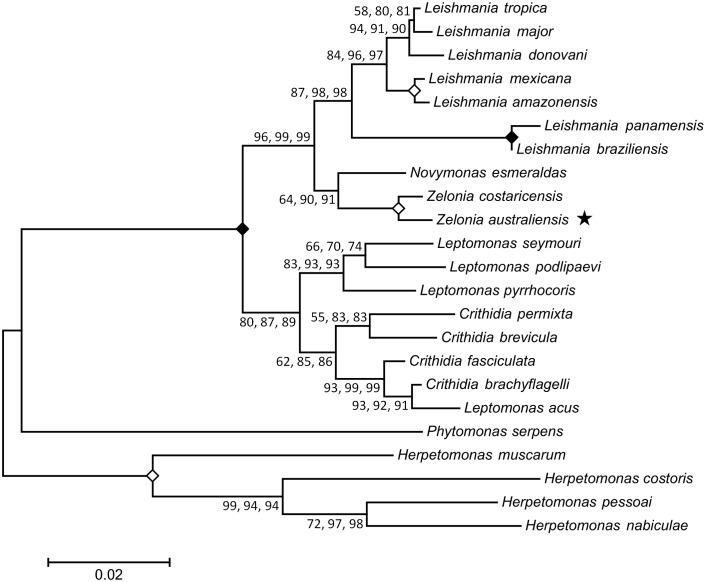
Inferred evolutionary relationship between *Zelonia australiensis* and other trypanosomatids using concatenated *18S rDNA* and *gGAPDH* sequences. This tree was constructed using sequences from 23 trypanosomatids, aligned to a total of 1302 positions with all gaps and missing data eliminated. The structure of this tree was inferred using three statistical methods; the ML method based on the Tamura-Nei model, the ME method [36] and the NJ method [37]. The same tree structure was predicted using each method. The first value at each node is the percentage of trees in which the associated taxa clustered together using the ML method (1000 replicates). The second and third number at each node is the percentage of replicate trees obtained for the ME and NJ methods respectively, in which the associated taxa clustered together in the bootstrap test (1000 replicates) [102]. A solid diamond indicates a node that obtained a value of 100% for all three methods. An open diamond indicates a node that obtained a value of at least 99% for each method. The star highlights the phylogenetic position of *Z*. *australiensis*. The bar represents the number of substitutions per site.

**Fig 7:**
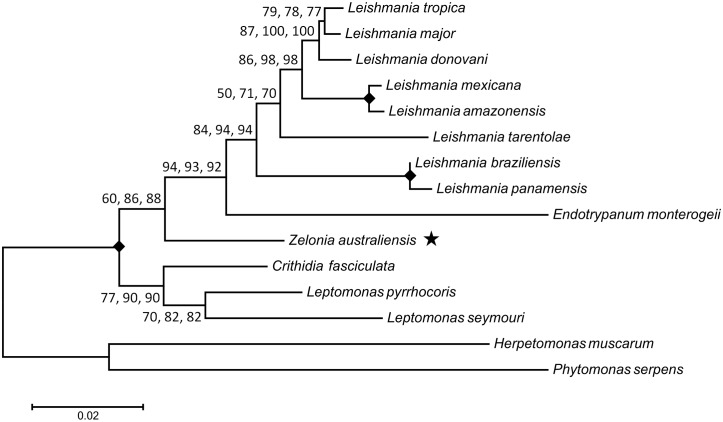
Inferred evolutionary relationship between *Zelonia australiensis* and other trypanosomatids using concatenated *18S rDNA*, *gGAPDH*, *RPOIILS* and *HSP70* sequences. This phylogenetic tree was constructed using sequences from 15 trypanosomatids, aligned to a total of 2344 positions with all gaps and missing data eliminated. The structure of this tree was inferred using three statistical methods; the ML method based on the Tamura-Nei model, the ME method [36], and the NJ method [37]. The same tree structure was predicted using each method. The first value at each node is the percentage of trees in which the associated taxa clustered together using the ML method (1000 replicates). The second and third number at each node is the percentage of replicate trees obtained for the ME and NJ methods respectively, in which the associated taxa clustered together in the bootstrap test (1000 replicates) [102]. A solid diamond indicates a node that obtained a value of 100% for all three methods. The star highlights the phylogenetic position of *Z*. *australiensis*. The bar represents the number of substitutions per site.

**Fig 8:**
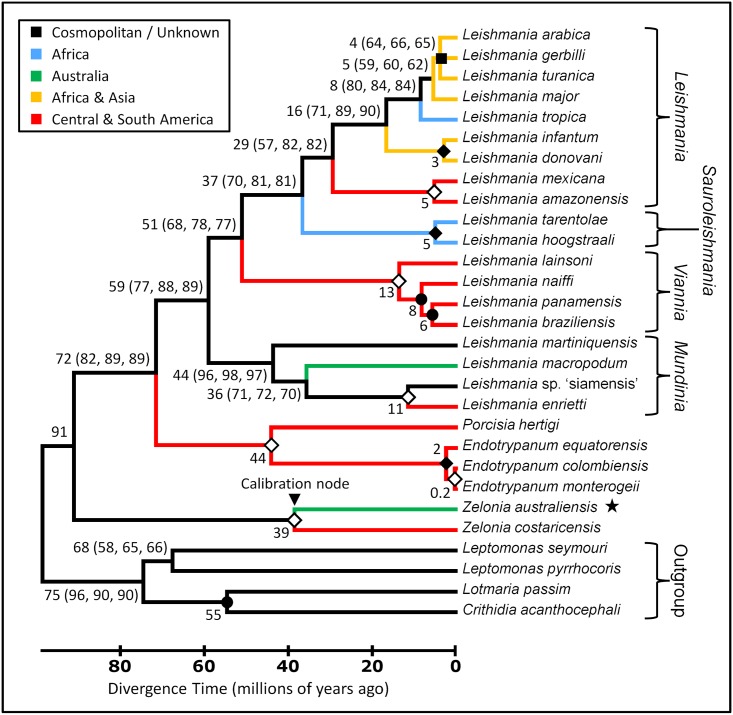
Phylogenetic Time Tree inferring the evolutionary relationships between *Zelonia australiensis* and other trypanosomatids using concatenated *18S rDNA* and *RPOIILS* sequences. This tree was constructed using sequences from 29 trypanosomatids, aligned to a total of 784 positions with all gaps and missing data eliminated. The structure of this tree was inferred using three statistical methods; the ML method based on the Tamura-Nei model, the ME method [36], and the NJ method [37]. The same tree structure was predicted using each method. The predicted minimum divergence times for each node i.e. the values outside the brackets, were predicted using the RelTime method [40]. Estimated divergence times greater than 1 MYA are rounded to the nearest whole number. The error calculated for the divergence time at each node is shown in S2 Fig. Regarding values within brackets, the first number is the percentage of trees in which the associated taxa clustered together using the ML method (1000 replicates). The second and third number is the percentage of replicate trees obtained for the ME and NJ methods respectively, in which the associated taxa clustered together in the bootstrap test (1000 replicates) [102]. An open diamond indicates a node that obtained a value of 99% or greater for each method. A solid diamond indicates a node that obtained a value of 100% for all methods. A solid circle represents nodes that obtained a value of 60% or less for each method. A solid square represents a collapsed node. The star highlights the phylogenetic position of *Z*. *australiensis*. Branches are colour coded to indicate the current dispersion pattern for the different species. Note that *Leishmania infantum* is also found in European countries flanking the Mediterranean basin. This time tree was calibrated by setting the node depicting the divergence of *Z*. *australiensis* and *Zelonia costaricensis* at 41 to 36 (mean ~39) million years ago; a minimum time estimate for the vicariance event that separated these taxa.

Phylogenetic trees were constructed from concatenated alignments of *18S rDNA* and *gGAPDH* sequences (Fig 6), and *18S rDNA*, *gGAPDH*, *RPOIILS* and *HSP70* sequences (Fig 7) to infer the phylogenetic relationship between this novel trypanosomatid and other related parasites. Concatenated sequence alignments were employed as these are generally considered more robust for inferring phylogenetic relationships [15]. For each alignment, phylogenies inferred using the ML, NJ and ME methods showed the same structure. Both phylogenies positioned this parasite in the subfamily Leishmaniinae, basal to the clade occupied by *Leishmania*, *Endotrypanum* and *Porcisia*. The phylogeny generated from the *18S rDNA* and *gGAPDH* concatenated sequence inferred *Z*. *costaricensis* as the sibling species to this new parasite, with a bootstrap percentage of at least 99, across 1000 replicates for each phylogenetic method used (ML, NJ and ME). Based on this result and the morphological characteristics previously described, this parasite was assigned to the genus *Zelonia* and will hereafter be referred to as *Zelonia australiensis* sp. nov. Once this classification was established, a phylogenetic time tree was constructed using concatenated sequences of the *18S rDNA* and *RPOIILS* genes, given that these phylogenetically informative sequences were available for many Leishmaniinae. The node representing the divergence of *Z*. *australiensis* and *Z*. *costaricensis* was selected as a calibration point. This node was set at 36 to 41 MYA which is the estimated time period that Australia and South America became completely separated [21], representing a minimum time for the separation of these taxa. Using this calibration point, an ancestor to *Leishmania*, *Endotrypanum* and *Porcisia* was predicted to have appeared approximately 91 MYA (Fig 8), inferring a Gondwanan origin for dixenous parasitism in the Leishmanaiinae subfamily [3]. Fig 8 also infers that the divergence of *Z*. *australiensis* from *Z*. *costaricensis*, and *Leishmania macropodum* from other *Mundinia* parasites occurred around the same time, just prior to the Eocene to Oligocene transition, which occurred between 33 and 34 MYA.

## Discussion

The genus *Leishmania* includes approximately 20 species of protozoan parasite that are the etiological agents of human leishmaniais [6], an important albeit neglected tropical disease. Relative to other protozoan diseases, leishmaniasis is second in importance to malaria as a cause of mortality [43], and WHO estimates suggest a disease burden of 2.35 million DALYs (Disability-Adjusted Life Years) lost as a result of leishmaniasis. *Leishmania* exists on all continents with the exclusion of Antarctica, though its geographical range is focused in the tropics and subtropics [6]. Despite Australia’s geographical isolation, representatives of this genus have also been found on this continent [44]. As a consequence of its wide global dispersion patterns, the biogeographical history of *Leishmania* has been hotly debated for decades and several hypotheses have been proposed.

The Palaearctic origins theory suggests that *Leishmania* originated in the Old World during the early Cenozoic period [8], and was later dispersed to the Nearctic and then the Neotropics via the Bering land bridge, which was open during the Eocene epoch but eventually closed approximately 33 to 35 MYA [6, 45]. The discovery of *P*. *proterus* fossilised in Burmese amber supports an Old World origin for *Leishmania*, though the age of the amber (100 to 110 million years old) supports an earlier Cretaceous origin [1, 9], consistent with recent phylogenies [3] (Fig 8). *Paleoleismania proterus* were visible in the proboscis of *Palaeomyia burmitis*, and amastigotes were noted in reptilian red blood cells within the fly [1, 9]. This led to the interpretation that a dixenous life cycle had evolved in the Leishmaniinae roughly 100 MYA in the Old World, and supported that Cretaceous reptiles were the first vertebrate hosts of the earliest dixenous Leishmaniinae [6, 8, 9]. However, this interpretation is not supported by current phylogenies that do not place the *Sauroleishmania* in a basal position or sister clade to all other *Leishmania* species [3, 46–49] (Fig 8).

While the fossilised forms identified within *P*. *burmitis* are compelling and undoubtedly represent an early trypanosomatid [1], inferring evolutionary relationships for protozoa based purely on morphology is precarious. Some of the forms described by Poinar and Poinar could easily represent epimastigotes of *Trypanosoma* spp. based on the location of the kinetoplast relative to the nucleus [1]. *Trypanosoma* spp. are basal to all Leishmaniinae and so a dixenous life cycle probably evolved in this genus much earlier [15]. Furthermore, *Trypanosoma* spp. are known to infect reptiles and some reptile-infecting trypanosomes are transmitted by sand flies [50–52]. Mixed trypanosomatid infections are also common in insects [53, 54], which further complicates interpretation of such evidence. Additionally, it is well established that trypanosomatids have undergone substantial molecular evolution despite minimal morphological change [55]. This phenomenon has led to erroneous taxonomic assignments, even for taxa that are presently alive today [15]. Consequently, assignment of these organisms to the Trypanosomatidae based on the fossil evidence at hand is warranted [1], though classifying these organisms at any greater resolution is probably tenuous.

The Neotropical origins hypothesis proposes that *Leishmania* evolved in South America between 34 and 46 MYA [3, 6]. Indeed, a Neotropical origin is supported by the evidence available, though the appearance of *Leishmania* probably occurred much earlier than the Neotropical hypothesis initially proposed (Fig 8) [3, 56]. The Neotropical origins theory is also supported by the limited range of the Paraleishmania which are restricted to the New World, and are basal to all Euleishmania [3, 6, 16] (Figs 7 and 8). The Multiple Origins hypothesis suggests the Euleishmania and Paraleishmania evolved on Gondwana prior to the opening of the Atlantic Ocean. When Africa and South America separated, the Euleishmania in the Old World evolved into the *Sauroleishmania* and *Leishmania* subgenera, while Euleishmania in the New World evolved into *Viannia* [3, 7]. A very ancient, African origin has been proposed for most Old World *Leishmania* species given their intimate relationships with certain rodent species and hyraxes; vertebrates that have a highly restricted range [7]. However, the results of this study and others suggest that the Old World *Leishmania* (*Leishmania*) parasites originated approximately 30 MYA [3] (Fig 8).

The present study supports a Gondwanan origin for dixenous parasitism in the Leishmaniinae subfamily, inferring the appearance of a common ancestor to the Euleishmania and Paraleishmania at approximately 91 MYA (Fig 8) [3]. This places the origin of the dixenous Leishmaniinae during the breakup of Gondwana when the radiation of mammals first began [57], and is within the lower limit of 90 to 140 MYA proposed recently by Harkins *et al*. [3]. By 90 MYA, Africa and South America had already separated. Multiple phylogenies suggest that *Viannia* emerged millions of years later (Fig 8) [3, 11, 57], implying that their divergence from other Euleishmania was not triggered by the separation of Africa and South America. The presence of the *Leishmania* subgenus in the New World is often discussed as a migration from the Old World to the New [58]. However, based on current evidence, an alternate scenario is proposed. Approximately 50 MYA the climate in the northern hemisphere was tropical and a series of land bridges, shallow seas and island chains connected Europe, North America and Asia [11, 59]. These land bridges were probably endemic for *Leishmania* and allowed movement of host and vector between the Old World and the New until approximately 35 to 33 MYA, when these bridges disappeared [45]. Paleontological evidence supports an exchange of primate and rodent species between North and South America during the same period, indicating that the Panamanian land bridge was also open [60]. The disappearance of these northern land bridges coincides with the sharp drop in temperature that signifies the Eocene to Oligocene transition [61]. This also coincides with the inferred emergence of the New World *Leishmania* (*Leishmania*) spp. approximately 30 MYA [3] (Fig 8). By 33 MYA, these once tropical northern land bridges were uninhabitable for sand flies, probably forcing the range of *Leishmania* and other tropical species south towards the Neotropics in the New World, and out of Northern Europe, towards Africa and South East Asia in the Old World. The presence of *L*. (*L*.) *amazonensis*/*mexicana* complex organisms in China supports this scenario [3, 62].

The subgenus *Mundinia* Shaw, Camargo and Teixeira 2016 was recently established to accommodate members of what was previously referred to as the *L*. *enrietti* complex [12]. While *Mundinia* are widely dispersed, *L*. (*M*.) *enrietti* itself was initially isolated from guinea pigs in Brazil and is probably native to the Neotropics [63]. A related organism, *Leishmania* (*Mundinia*) *martiniquensis*, was later identified on the Caribbean Island of Martinique, detected in immunocompromised patients presenting with cutaneous leishmaniasis (CL) and visceral leishmaniasis (VL) [64–66]. Parasites of the *Mundinia* subgenus have since been identified in Thailand i.e. *Leishmania* sp. 'siamensis', as a cause of human VL, predominantly in immunosuppressed patients [67–70]. As discussed by other investigators [46], *Leishmania* 'siamensis' represents a *nomen nudum*, and is shown inverted commas here as a consequence. *Leishmania* 'siamensis' was detected in horses from the USA and central Europe [71, 72], and in Swiss cows [73]. The geographical range of *L*. 'siamensis' and *L*. (*M*.) *martiniquensis* is known to overlap given the recent detection of *L*. (*M*.) *martiniquensis* in Thailand [46], resulting in misidentification in some cases [46, 74]. Additionally, a unique *Mundinia* parasite was only recently identified as a cause of human CL in Ghana [46], though this organism is yet to be named. *Leishmania* (*M*.) *macropodum* is also a member of the *Mundinia* subgenus, and is recognised as a cause of CL in Australian native macropods [44, 75].

The global dispersion pattern of *Mundinia* is difficult to explain, though the current range of *L*. (*M*.) *martiniquensis* may be related to human activities such as international shipping and trade, facilitating the movement animals i.e. livestock, companion animals and rodents, between regions that would have otherwise been non-traversable. Indeed, rats have been pivotal to the global dispersion of other parasites via this route [76]. Furthermore, *Mundinia* parasites are not necessarily restricted to sand fly vectors, which could facilitate their adaptation to new regions [20, 22]. As a consequence of these dispersion patterns, it is difficult to infer where *Mundinia* originally appeared.

Current phylogenies suggest that the Ghanian parasite and *L*. *enrietti* diverged within the last 10 million years [3, 46]. These species have been observed in only a few restricted regions implying that their native range is limited. Perplexingly, this suggests that these two parasites diverged long after the New World separated from Africa. During the Miocene epoch there was a warm period in central Europe which abruptly ended at ~14 MYA, when temperatures dropped markedly to a mean annual temperature of ~14.8°C to15.7°C [77, 78]. Consequently, it is unlikely that movement of *Leishmania* between the Nearctic and Palearctic occurred via the northern land bridges at this time. Alternatively, dispersion of an ancestral *Mundinia* parasite between the Old World and the New as recently as 10 MYA may have been facilitated by far-travelling marine mammals (seals), or bats, which are potential hosts of *Leishmania* [79–83]. Alternatively, recent satellite evidence has revealed a scattering of numerous seamounts across the Atlantic Ocean [84]. At 10 MYA, these seamounts may have existed as a large volcanic island chain that allowed movement of terrestrial organisms across the Atlantic, but eventually eroded into the sea [85]. However, it should be noted that these possibilities are purely speculative and not well supported by the evidence at hand.

Australia was considered free of *Leishmania* until the discovery of *L*. (*M*.) *macropodum* in 2004 [44]. Prior to the present study *L*. (*M*.) *macropodum* had not been formally described. Therefore, the name it was informally assigned i.e. *Leishmania* ‘australiensis’, represents a *nomen nudum*. However, the formal description provided herein resolves this issue. Based on current evidence, the presence of *L*. (*M*.) *macropodum* in Australia is most likely the result of vicariance; the complete separation of Australia from South America by roughly 40 MYA [3, 21]. This study infers that the divergence of *Z*. *australiensis* from *Z*. *costaricensis*, and *L*. (*M*.) *macropodum* from other *Mundinia* parasites, occurred within approximately 3 million years of each other, approaching the Eocene to Oligocene transition (Fig 8). Given the margins of error associated with such predictions (S2 Fig) and the concurrence between the inferred divergence times of these taxa, the estimates presented here are plausible. This scenario is also consistent with the biogeography of other taxa, including the distribution of the plant genus *Nothofagus* and that of marsupials, which are generally restricted to parts of Central and South America, Australia and Oceania [3, 86].

*Novymonas esmeraldas*, *Z*. *costaricensis* and *Z*. *australiensis* are presumably monoxenous trypanosomatids basal to all dixenous Leishmaniinae (Fig 6) [4, 16], and probably represent the nearest ancestors of a parasite that transitioned from a monoxenous to a dixenous life cycle [87]. The rigorous growth of *Z*. *australiensis* in high haemoglobin concentrations and on chocolate agar is consistent with a haemoprotozoan (Fig 2, S1 File) [88] and/or adaptation to life as a parasite of hematophagous insects, which probably represents the first step in the transition to a dixenous life cycle. While *Z*. *costaricensis* was originally isolated from a non-hematophagous reduviid bug, *Ricolla simillima*, these insects are predatory and may have recently fed on a hematophagous insect prior to the isolation of *Z*. *costaricensis* [89]. This is conceivable as *Novymonas* which was first isolated and described from *Niesthrea vincentii* (Hemiptera: Rhopalidae) has also been detected in *Zelus* sp. (an assassin bug) and *Culicoides* sp. (a hematophagous midge) [16].

As parasites occupying the *Novymonas*/*Zelonia* clade (Fig 6) infect varied and disparate hosts, it is difficult to infer their vicariance based on host distribution. Also, given the origins of the Australian Simuliidae, their role in the dispersion of *Zelonia* is probably limited. Dumbleton [90] suggested that *Simulium* entered Australia from the north during what was then referred to as the Tertiary period, between 65 and 1.6 MYA. Similarly Crosskey [25] was of the firm opinion that *Simulium* entered Australia from the north via a land bridge that once connected Australia and New Guinea, but no time was suggested. As Australia drifted north, the interaction of New Guinea as the leading edge to the Australian Plate with the Pacific Plate and others, was complex and is discussed in some detail by Craig *et al*. [91] in relation to formation of the Solomon Islands. Given the distribution of various segregates of *Simulium*, colonization of this genus into New Guinea could have occurred as early as the mid Eocene to early Miocene (20 to 40 MYA). *Simulium dycei* is a member of subgenus *Morops* that is centred and diverse in New Guinea, an indication it is an older segregate of *Simulium* that colonized this land mass originally. A good assumption would be that *Simulium* has been on the Australian land mass for 40 MYA at most [91].

Despite the concurrence between the inferred arrival dates of *Simulium* in Australia and the appearance of *Z*. *australiensis*, it is unlikely that *Zelonia* was dispersed from South America to Australia via the Nearctic, the Palearctic and then South East Asia to arrive with *Simulium*. If dispersion of Leishmaniinae via this route occurred during this period, one might expect to encounter close relatives of *L*. (*M*.) *macropodum* or other dixenous species in Papua New Guinea, the Solomon Islands and/or parts of Indonesia, though no such reports exist. Consequently, the available evidence suggests that the separation of Australia from South America gave rise to *Z*. *australiensis* and *L*. (*M*.) *macropodum*. *Zelonia* probably came to infect *Simulium* when this genus arrived from New Guinea around 40 MYA. Prior to this, *Zelonia* was likely already in Australia, parasitizing other insect species. Indeed, investigation of other Australian insects such as native reduviids and *Culicoides* spp. for infection with *Z*. *australiensis* is warranted.

*Leptomonas* spp. are considered monoxenous parasites that are generally of no clinical importance [92–94]. However, *L*. *seymouri*, originally isolated from the phytophagous cotton stainer bug, *Dysdercus suturellus* [95], is capable of infecting humans opportunistically, inducing co-infections with *L*. (*L*.) *donovani* [96, 97]. Its ability to cause human infections implies that *L*. *seymouri* also possesses an alternate hematophagous host [98]. While they are still considered monoxenous, and are continually grouped in basal clades to *Leishmania* [16, 17, 99] (Figs 6, 7 and 8), it is plausible that certain monoxenous Leishmaniinae are ancestors of transitional forms that did not complete the switch to a dixenous life cycle. Indeed, monoxenous trypanosomatids occasionally explore the dixenous niche based on multiple reports of infections involving animals and humans [98]. Genome sequencing and transcriptome profiling identified several adaptations in *L*. *seymouri* that allow it to persist in the vertebrate host environment [100]. Furthermore, *L*. *seymouri* survived for several days in two species of phlebotamine sand fly [100]. Given their close relationship with *Leishmania*, *Leptomonas* spp. represent interesting models for studying the transition from a monoxenous to dixenous life cycle, including the evolutionary innovations that enable parasitism of vertebrate hosts [98, 100]. Moreover, the ability of *L*. *seymouri* to infect humans under some circumstances raises questions as to whether *Novymonas* and *Zelonia* are truly monoxenous, or if they might also be capable of infecting vertebrates under some circumstances, occasionally exploring the dixenous niche.

To conclude, we described the first isolation of *Zelonia australiensis* sp. nov. from the Australian native black fly *S*. (*M*.) *dycei* in Australia’s Northern Territory. A detailed molecular and morphological characterisation was performed to establish this assignment, including light and electron microscopy, sequencing and phylogenetic analyses. As a result, *Z*. *australiensis* was identified as a sibling taxon to the monoxenous trypanosomatid, *Z*. *costaricensis*. Subsequently, the divergence of these species was used as a unique calibration point for a phylogenetic time tree exploring the relationships between several species of the Leishmaniinae subfamily. These analyses inferred the emergence of dixenous parasitism in the Leishmaniinae at approximately 91 MYA, in Gondwana, during the Cretaceous period. Ultimately, this study contributes to our understanding of trypanosomatid diversity by describing a unique Australian species, and to our understanding of *Leishmania* evolution by providing support for a Gondwanan origin of dixenous parasitism in the Leishmaniinae.

### Taxonomic summary for *Zelonia australiensis*

**Class:** Kinetoplastea Honigberg, 1963 emend. Vickerman, 1976
**Subclass:** Metakinetoplastina Vickerman, 2004
**Order:** Trypanosomatida Kent, 1880
**Family**: Trypanosomatidae Doflein, 1901
**Subfamily:** Leishmaniinae Maslov and Lukes 2012 emend. Shaw, Camargo and Teixeira 2016 [12]
**Genus:** *Zelonia* Shaw, Camargo and Teixeira 2016 [12]
**Species:** *Zelonia australiensis* Barratt, Kaufer and Ellis 2017
**Species diagnosis:** A trypanosomatid of the genus *Zelonia* morphologically and ultrastructurally similar to *Zelonia costaricensis* (previously *Leptomonas costaricensis* [4]) (Figs 3 and 4, S2 File), which is its sibling taxon. When cultured axenically, individuals of *Zelonia australiensis* exist in a variety of morphotypes as detailed in Fig 3. The species is also defined by a set of unique sequences of the *18S rDNA*, *gGAPDH*, *RPOIILS*, *HSP70* and ITS1 (GenBank Accessions: KY273490 to KY273515).
**Etymology**: The species name is derived from the country Australia, where the organism was first isolated.
**Type host:** Originally isolated from pooled specimens of female *Simulium* (*Morops*) *dycei*, Colbo 1976 (Diptera: Simuliidae) (Fig 1, S1 Fig).
**Type locality:** Vicinity of Darwin, Northern Territory, Australia. The precise coordinates of isolation are provided in Table 1.
**Type material:** Axenic cultures are currently maintained at the University of Technology Sydney, Ultimo, NSW, Australia. Cryogenically frozen material is also stored at this location.

### Taxonomic summary for *Leishmania* (*Mundinia*) *macropodum*

**Class:** Kinetoplastea Honigberg, 1963 emend. Vickerman, 1976
**Subclass:** Metakinetoplastina Vickerman, 2004
**Order:** Trypanosomatida Kent, 1880
**Family**: Trypanosomatidae Doflein, 1901
**Subfamily:** Leishmaniinae Maslov and Lukes 2012 emend. Shaw, Camargo and Teixeira 2016 [12]
**Genus:** *Leishmania* Ross, 1903
**Subgenus:** *Mundinia* Shaw, Camargo and Teixeira 2016 [12]
**Species:** *Leishmania* (*Mundinia*) *macropodum* Barratt, Kaufer and Ellis 2017
**Species diagnosis:** The species is defined by the detailed descriptions and images provided by Rose *et al*. [44] and Dougall *et al*. [20, 75], and by a set of DNA sequences accessible in GenBank: HM775497.1, AY495831.1, AY495830.1, AY495829.1 and FR693774.2.
**Type strain:** *Leishmania* sp. AM-2004/*Leishmania* sp. Roo1
**Etymology**: The species name is derived from the only known vertebrate hosts of this parasite which includes several species of marsupial from the family Macropodidae.
**Type host:** The parasite was first isolated from a red kangaroo, *Osphranter rufus* [44], though natural vertebrate hosts of *L*. (*M*.) *macropodum* include several other species of Australian macropod [75].
**Type vector:** *Forcipomyia* (*Lasiohelia*) spp. midges are the likely vector [20], although experimental infections have been achieved in *Culicoides* midges [22].
**Type locality:** Vicinity of Darwin, Northern Territory, Australia.
**Type Material:** For information on type material contact the investigators who first identified and later isolated *L*. (*M*.) *macropodum* [20, 44, 75].
**Remarks:** Barratt *et al*. take no credit for the discovery or isolation of *L*. (*M*.) *macropodum*. This parasite has been referred to as *Leishmania* ‘australiensis’ in previous works in the absence of any formal description [6, 23], making it a *nomen nudum* and consequently unavailable for future use. This parasite was formally described as *L*. (*M*.) *macropodum* herein, simply to avoid the continued use of a *nomen nudum*.

## Supporting Information

S1 Fig Morphology of female *Simulium* (*Morops*) *dycei*, Colbo 1976 exoskeletons (flies A and B) following DNA extraction. This figure shows exoskeletons from two black flies (designated as Fly A and Fly B) following DNA extraction for downstream PCR. (A) Habitus of *S*. (*M*.) *dycei* female (Fly B) in Euparal mounting media. (B) Mandible of *S*. (*M*.) *dycei* female, serrated on both edges (Fly A). (C) Genital fork of *S*. (*M*.) *dycei* female with strongly sclerotized shaft and basal arm (Fly A). (D) Haired anepisternal (pleural) membrane of *S*. (*M*.) *dycei* female (Fly A). Few hairs are present on this specimen due to damage caused during specimen preparation and DNA isolation, indicated by numerous pores at the site of setal insertion. (E) Antenna of *S*. (*M*.) *dycei* female consisting of 11 segments with 3 basal segments paler in colour compared to the apical segments (Fly A). (F) Wing of *S*. (*M*.) *dycei* female with small dark spinules along costa and distally on radius, both veins are haired (Fly A). (G) Hind leg tarsomeres of *S*. (*M*.) *dycei* female showing the well-developed pedisulcus and calcipala. The claw lacks a basal tooth (Fly A). This figure confirms that the fly-derived PCR products generated in this study are indeed from two individuals of *S*. (*M*.) *dycei*. Sequences obtained for the *COI*, *COII*, *18S rRNA* and *28S rRNA* genes from flies A and B are available in GenBank (Accession numbers KY288010 to KY288017).
(TIF)

S2 Fig Phylogenetic time tree with error bars, inferring the evolutionary relationships between *Zelonia australiensis* and other trypanosomatids using concatenated *18S rDNA* and *RPOIILS* sequences. This Supplementary Figure shows the same phylogenetic tree displayed in Fig 8, though with error bars provided at each node, and estimated divergence times indicated. Estimated divergence times greater than 1 MYA are rounded to the nearest whole number. The star highlights the phylogenetic position of *Z*. *australiensis*.
(TIF)

S1 File Supplementary materials and methods. This file provides greater detail on several of the methods employed in this study.
(DOCX)

S2 File Footage of a motile promastigote of *Zelonia australiensis* under phase contrast microscopy. This footage shows a single typical promastigote cultured in NNN medium immediately after its isolation from *S*. (*M*.) *dycei* i.e. before passaging. This specimen represents one of the more common promastigote forms of the parasite. The typical, rapid, whip-like movement of the flagellum is apparent.
(AVI)

S1 Table Sequences used in phylogenetic analyses. This table lists the GenBank accession numbers for all nucleotide sequences used to construct phylogenetic trees in this study.
(DOCX)
