# Supplementary figures and images for: Isolation of Novel Trypanosomatid, Zelonia australiensis sp. nov. (Kinetoplastida: Trypanosomatidae) Provides Support for a Gondwanan Origin of Dixenous Parasitism in the Leishmaniinae
Source: PLoS Negl Trop Dis. 2017 Jan 12;11(1):e0005215. doi: 10.1371/journal.pntd.0005215 (PMC5230760; doi:10.1371/journal.pntd.0005215)

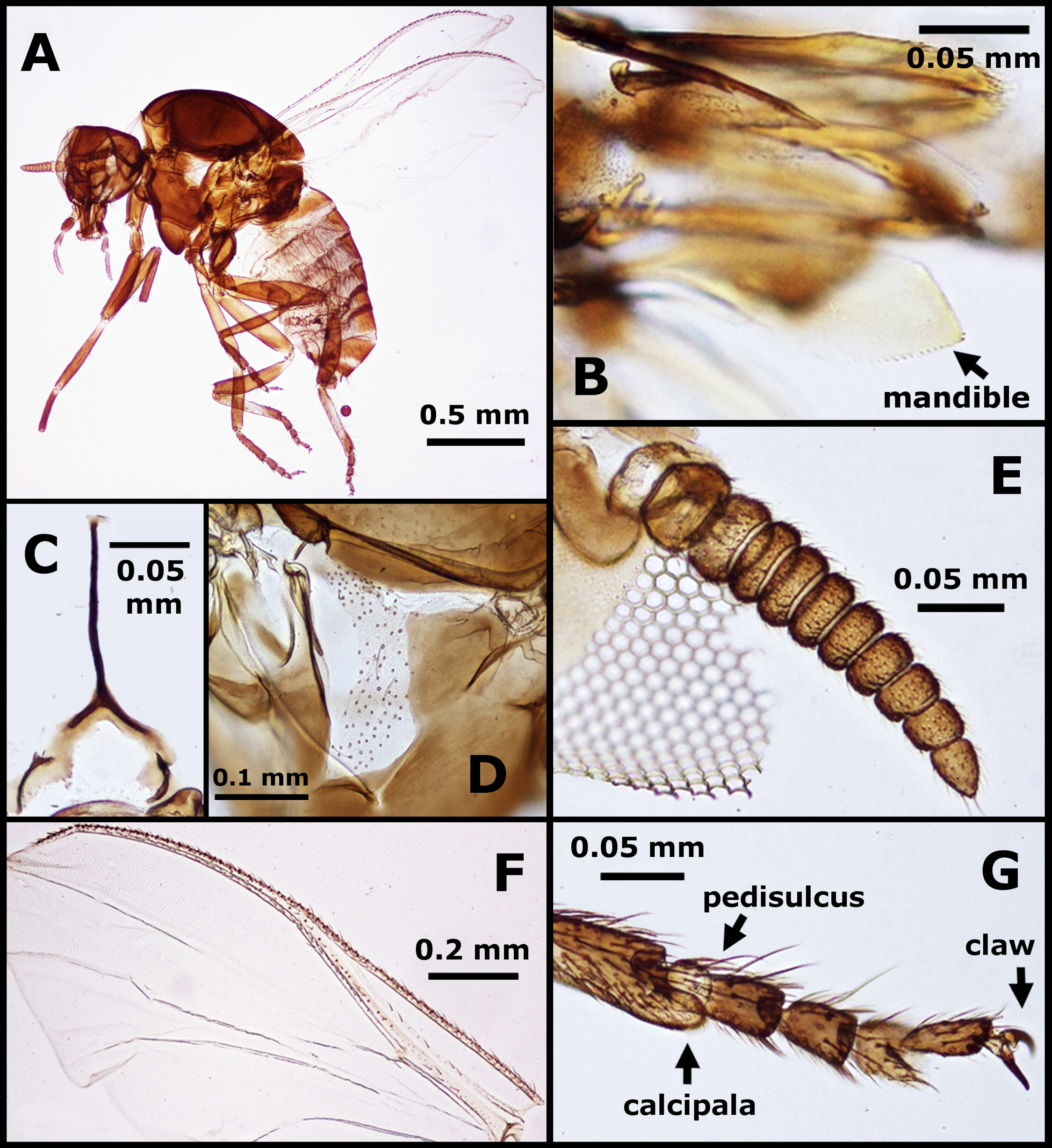

Supplement: S1 Fig — This figure shows exoskeletons from two black flies (designated as Fly A and Fly B) following DNA extraction for downstream PCR. (A) Habitus of S. (M.) dycei female (Fly B) in Euparal mounting media. (B) Mandible of S. (M.) dycei female, serrated on both edges (Fly A). (C) Genital fork of S. (M.) dycei female with strongly sclerotized shaft and basal arm (Fly A). (D) Haired anepisternal (pleural) membrane of S. (M.) dycei female (Fly A). Few hairs are present on this specimen due to damage caused during specimen preparation and DNA isolation, indicated by numerous pores at the site of setal insertion. (E) Antenna of S. (M.) dycei female consisting of 11 segments with 3 basal segments paler in colour compared to the apical segments (Fly A). (F) Wing of S. (M.) dycei female with small dark spinules along costa and distally on radius, both veins are haired (Fly A). (G) Hind leg tarsomeres of S. (M.) dycei female showing the well-developed pedisulcus and calcipala. The claw lacks a basal tooth (Fly A). This figure confirms that the fly-derived PCR products generated in this study are indeed from two individuals of S. (M.) dycei. Sequences obtained for the COI, COII, 18S rRNA and 28S rRNA genes from flies A and B are available in GenBank (Accession numbers KY288010 to KY288017). (TIF) [file pntd.0005215.s001.tif]

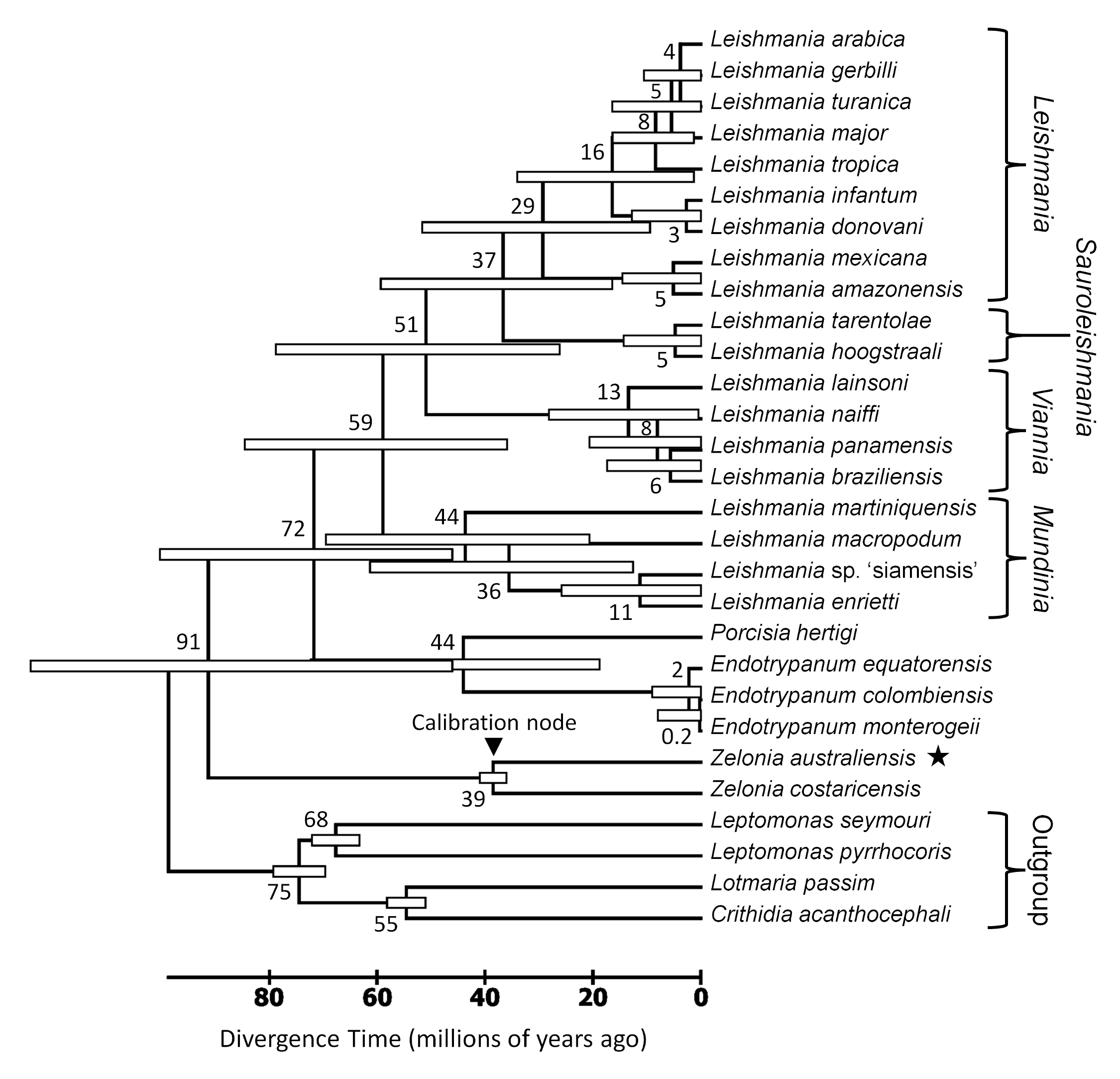

Supplement: S2 Fig — This Supplementary Figure shows the same phylogenetic tree displayed in Fig 8, though with error bars provided at each node, and estimated divergence times indicated. Estimated divergence times greater than 1 MYA are rounded to the nearest whole number. The star highlights the phylogenetic position of Z. australiensis. (TIF) [file pntd.0005215.s002.tif]
