# Supplementary material for: Isolation of Novel Trypanosomatid, Zelonia australiensis sp. nov. (Kinetoplastida: Trypanosomatidae) Provides Support for a Gondwanan Origin of Dixenous Parasitism in the Leishmaniinae
Source: PLoS Negl Trop Dis. 2017 Jan 12;11(1):e0005215. doi: 10.1371/journal.pntd.0005215 (PMC5230760; doi:10.1371/journal.pntd.0005215)
Supplement: S1 File — This file provides greater detail on several of the methods employed in this study. (DOCX) [file pntd.0005215.s003.docx]

**Supplementary File 1.**

**This file provides a set of detailed materials and methods as a supplement to the following manuscript:**

Isolation of Novel Trypanosomatid, *Zelonia australiensis* sp. nov. (Kinetoplastida: Trypanosomatidae) Provides Support for a Gondwanan Origin of Dixenous Parasitism in the Leishmaniinae

Joel Barratt^1^, Alexa Kaufer^1^, Bryce Peters^1,2^, Douglas Craig^3^, Andrea Lawrence^4,5^, Tamalee Roberts^6^, Rogan Lee^7^, Gary McAuliffe^8^, Damien Stark^6^, John Ellis^1^

^1^ School of Life Sciences, University of Technology Sydney, NSW, Australia

^2^ Insect Research Facility, University of Technology Sydney, NSW, Australia

^3^ Department of Biological Sciences, University of Alberta, Edmonton, Alberta, Canada

^4^ Faculty of Veterinary Science, University of Sydney, New South Wales 2006, Australia

^5^ Department of Medical Entomology, University of Sydney & Pathology West - ICPMR, Westmead Hospital, Westmead, NSW, Australia

^6^ St. Vincent's Hospital Sydney, Division of Microbiology, NSW, Australia

^7^ Centre for Infectious Diseases and Microbiology Laboratory Services, ICPMR, Westmead Hospital, Westmead, NSW, Australia

^8^ Microbiology Department, Royal Darwin Hospital, Darwin, Northern Territory, Australia

**TABLE OF CONTENTS**

[Study location and insect trapping 1](#_Toc469330288)

[Insect identification and sorting 1](#_Toc469330289)

[Cultivation of parasites 2](#_Toc469330290)

[Axenisation of promastigote cultures 2](#_Toc469330291)

[Effect of haemoglobin on promastigote growth 3](#_Toc469330292)

[Transmission electron microscopy 4](#_Toc469330293)

[Extraction of genomic DNA from insects to conserve their exoskeleton 6](#_Toc469330294)

[Polymerase Chain Reaction (PCR) 6](#_Toc469330295)

[Restriction fragment length polymorphism (RFLP) analysis 7](#_Toc469330296)

[Direct sequencing of PCR amplicons 7](#_Toc469330297)

[Sequencing of cloned PCR amplicons 8](#_Toc469330298)

# Study location and insect trapping

Insects were trapped in the Northern Territory Wildlife Park, Berry Springs, Australia, approximately 42 kilometres south of Darwin. John W. Hock CDC Miniature light traps (Model 512) were set in a macropod enclosure that housed *Macropus antilopinus* (Antilopine Wallaroos). The CO_2_ regulators on traps were set for an output of 200 mL of CO_2_ per minute. Trapping was performed on the 27^th^ of February in 2014, during Darwin’s wet season and took place in two sessions; from approximately 10 am to 12 noon, and from approximately 12 noon to 2 pm. These sessions were restricted to two hours to prevent desiccation of trapped insects which could reduce parasite viability. Three trap sites were selected within the enclosure and two traps were set at each site. Insects were also aspirated directly from the fur of captive Antilopine Wallaroos and *Macropus agilis* (Agile Wallabies) housed in or near the enclosures mentioned above. This was performed using small, hand-held, battery powered aspirators. Following trapping and aspiration, insect trap bags were covered with a moist cloth and placed in a plastic cooler box to prevent insect desiccation. Insects were transported to the Medical Entomology Department at Royal Darwin hospital for identification and sorting.

# Insect identification and sorting

Trap bags were placed at 4°C for approximately 5 minutes to immobilise the insects. Insects were emptied onto a cooling block (4°C) and identified with the aid of a magnifier. In an attempt to reisolate *L.* (*M.*) *macropodum*, *Forcipomyia* (*Lasiohelea*) spp., Kieffer 1921, were identified by dichotomous key. During insect collection, a species of black fly (Family: Simuliidae) was frequently observed biting macropods and was abundantly recovered from insect traps. Consequently, these Simuliidae were selected for further study. Several black fly specimens were dissected and mounted, and identified as *Simulium* (*Morops*) *dycei* using descriptions in the Zoological Catalogue of Australia, and by dichotomous key. Specimens of *Forcipomyia* (*L.*) spp. and *S*. (*M*.) *dycei* were also subjected to parasite culture. As no sequence data was available for *S*. (*M*.) *dycei* in GenBank at the time, DNA was extracted from flies for PCR and sequencing. To ensure assignment of sequences to the correct insect species, DNA was extracted using a method that conserved the fly exoskeleton for later morphological confirmation (described below). Following DNA extraction, fly exoskeletons were clarified in 10% KOH for 30 minutes and then washed with distilled water. The exoskeletons were dehydrated with an ethanol series (70%, 80%, 95% and 100%) for an hour at each concentration and then slide mounted in Euparal mounting media (Australian Entomological Supplies, Coorabell Australia) for taxonomic identification. The salient structures, including the genitalia (genital fork) were dissected from a specimen and mounted for morphological identification with the aid of keys and descriptions.

# Cultivation of parasites

*Forcipomyia* (*L.*) spp. or *S*. (*M*.) *dycei* were pooled (10 to 20 specimens of the same species) and crushed with a spatula in ~200 µL of PBS. The resulting suspension was used to inoculate a *Leishmania* culture medium. This medium consisted of a Novy, McNeil, Nicolle (NNN) defibrinated rabbit blood slope overlaid with a liquid phase containing single strength penicillin-streptomycin (Gibco), 250 µg/mL gentamycin (Sigma-Aldrich), 2.5 µg/mL hemin (Sigma-Aldrich), 2.5 µg/mL biopterin (Sigma-Aldrich) in single strength M199 (pH 7) (Sigma-Aldrich), with 10% heat inactivated foetal bovine serum (Gibco). Cultures were incubated at ambient room temperature in the Microbiology Department at Royal Darwin Hospital, and examined by wet mount for promastigotes every two days for approximately 2 weeks.

# Axenisation of promastigote cultures

Promastigote cultures were initially contaminated with a *Fusarium* sp. fungus. Attempts were made to axenise cultures by streaking them onto Chocolate, Horse Blood (HB), and Sheep Blood (MH + SB) Agar plates (Sigma-Aldrich), and by the pour plate technique, to allow isolation of pure promastigote colonies. Plates were incubated at room temperature for 7 days before examination for parasite colonies. Attempts were also made to axenise cultures by serial dilution. Briefly, the liquid overlay from a fungi-contaminated promastigote culture was serially diluted tenfold, in the sterile liquid overlay described above, to dilutions ranging from 1:20 to 1:2 million. This was performed in 1 mL volumes, supplemented with ~0.5 g of chocolate agar, sliced from pre-poured chocolate agar plates with a sterile scalpel. Cultures were incubated at room temperature for 7 days and then examined for the presence of fungi and/or promastigotes by wet mount microscopy.

Promastigote colonies were absent on HB and MH + SB agar after 7 days incubation. In contrast, promastigotes grew rigorously on chocolate agar though the swarming growth of the *Fusarium* sp. fungus meant that pure parasite colonies were not obtained. However, an axenic promastigote culture was achieved at a serial dilution of 1:2 million. When struck onto chocolate agar plates, promastigotes produced translucent, irregular, swarming colonies that appeared after approximately 7 days incubation at ambient room temperature. Given the rigorous growth of promastigotes on chocolate agar compared to the NNN blood slopes (unpublished observations) the NNN slopes were replaced with chocolate agar. The biphasic medium was later abandoned in favour of a purely liquid medium (M3, see below) which contains the key components of chocolate agar (namely haemoglobin and IsoVitaleX), without the agar. While the biphasic medium produced excellent growth, the liquid medium was favoured for routine cultivation as it was more conducive to downstream counting experiments.

# Effect of haemoglobin on promastigote growth

Three solutions of differing haemoglobin concentration (0.02 g/L, 1 g/L and 2 g/L) were prepared in ddH_2_O using powdered bovine haemoglobin (BD BBL), and autoclaved at 121°C for 15 minutes. Once cooled, 500 mL of each haemoglobin solution was added aseptically to 500 mL of complete M199; pH 7, with 10% heat inactivated horse serum (Bovogen Biologicals) and single-strength penicillin-streptomycin, sterile filtered. Finally, 10mL of IsoVitaleX (BD BBL) was aseptically added to each solution to a final volume of 1.01 L. This gave rise to three media formulations with different haemoglobin concentrations; 0.0099 g/L (designated as M1), 0.495 g/L (M2) and 0.99 g/L (M3). A solution of complete M199 + 9.9mL/L Isovitalex, without haemoglobin, was included as a negative control (M0). Axenic promastigotes were grown at room temperature in a biphasic medium containing complete M199 overlaid on a solid phase of chocolate agar, for use as the inoculum (N0) for each of the different media (M0 to M3). After 48 hours incubation at room temperature, quantification of promastigotes in N0 was performed using KOVA slide counting chambers (KOVA, CA, USA) according to the manufacturer's instructions. As promastigotes are highly motile and difficult to count, 1 mL of N0 was transferred to a 1.5 mL tube and three drops of sodium acetate-acetic acid-formalin (SAF) was added. The solution was inverted several times, rendering the promastigotes immotile. The promastigote suspensions were then quantified using KOVA slide counting chambers. Following N0 quantification, twelve 50 mL falcon tubes were prepared; three containing 50 mL of each M0, M1, M2 and M3, such that the experiment was performed in triplicate. A single microliter of N0 (~1900 promastigotes) was used to inoculate the 12 tubes. Quantification of promastigotes in each tube began the following day (day 1) and was performed over a period of six days.

# Transmission electron microscopy

Saturated promastigote cultures were collected in 1.5 mL tubes and centrifuged at 300 g for 20 minutes. The supernatant was removed and promastigotes were fixed by suspension in electron microscopy grade fixative; 5% gluteraldehyde in phosphate buffered solution (0.1 M, pH 7.2). Fixation was carried out overnight at 4°C. The fixed promastigotes were centrifuged at 300 g for 20 minutes and the supernatant removed. The promastigotes were washed twice in electron microscopy grade 0.1 M sodium cacodylate buffer. Following washing, promastigotes were embedded in 1% low-melting temperature agarose. Firstly, 2 g of UltraPure Low Melting Point Agarose powder (Life Technologies) was added to 200 mL of boiling phosphate buffered saline which was then allowed to cool to 45°C. At this temperature a few small drops of agar were transferred to the tube containing the fixed promastigotes using a transfer pipette, and the tube shaken gently to suspend the cells. The agarose was then allowed to set at room temperature. Agarose blocks were cut into ~1 mm cubes with a single-edged razor. These cubes were then fixed in a solution of 50:50 2% osmium tetroxide & 0.2 M sodium cacodylate (pH 7.2) for 60 minutes. The cubes were then washed in buffered 0.1 M sodium cacodylate (pH 7.2) for 15 minutes, then in distilled water for 15 minutes. This was followed by a second post-fixation in a 50:50 solution of saturated uranyl acetate and distilled water for 60 minutes. The cubes were then washed in distilled water for 15 minutes. Next, the cubes were dehydrated by stirring in research grade ethanol in a series of increasing ethanol concentrations as follows: 25% ethanol – 2 minutes, 50% - 2 minutes, 70% - 2 minutes, 95% - 2 minutes, 100% - 15 minutes and 100% for another 15 minutes. The cubes were transferred to an analytical reagent grade acetone for two changes of 15 minutes each, and then placed in a 50:50 solution of acetone and Spurr’s resin for 30 minutes. The acetone/Spurr’s resin solution was removed and the cubes infiltrated with 100% Spurr’s resin for 2 to 3 hours, followed by curing at 58°C for 15 hours. Finally, ultrathin sections were cut onto grids, and then stained for 10 minutes in saturated uranyl acetate, followed by 5 minutes in Reynolds lead citrate. Ultrathin sections were examined using a Hitachi H-7650 Transmission Electron Microscope (USA).

# Extraction of genomic DNA from insects to conserve their exoskeleton

For extraction of genomic DNA from flies whilst preserving their exoskeleton, DNA extractions were performed using an Isolate II Genomic DNA Kit (Bioline) with the following modifications: whole flies were placed in 180 µL of lysis buffer and 25 µL of Proteinase K was added. The tube was incubated overnight at 56°C. For flies selected for testing by Leishmaniinae-specific PCR (described below), a small incision was made in the abdomen of flies using a sterile scalpel, prior to placing the whole specimen in lysis buffer. This would ensure thorough digestion of the flies’ internal organs where the parasites reside. Fly exoskeletons were carefully removed from the resulting digest solution using sterile tweezers and placed in 70% ethanol at -20°C for later species confirmation. The digest solution was subjected to DNA extraction according to the manufacturer’s instructions.

# Polymerase Chain Reaction (PCR)

PCR primers were designed to amplify and sequence phylogenetically relevant parasite genes. This was done firstly by aligning trypanosomatid sequences already available in GenBank. Highly conserved regions were identified from these alignments and used as the template for primer design, so that the resulting primers would produce a PCR product for all parasites of the subfamily Leishmaniinae. In this way, primers were designed for the *18S rRNA* gene and three protein coding genes; the glycosomal glyceraldehyde 3-phosphate dehydrogenase (*gGAPDH*), RNA polymerase II largest subunit (*RPOIILS*), and heat shock protein 70 (*HSP70*) genes. To generate sequence data for the black fly, a set of previously published primers were used to amplify fragments of the cytochrome C oxidase subunit I (*COI*) and II (*COII*) genes, the *18S rRNA* gene, and the *28S rRNA* gene (refer to the full manuscript for details). Each PCR was prepared from reagents provided in a BIOTAQ PCR Kit (Bioline). Reactions contained 2.5 U of BIOTAQ DNA Polymerase (5 U/μL), 4 µL of 10 mM dNTPs, 5 µL of 10 strength NH_4_ reaction buffer, 2 µL of 50 mM MgCl_2_, 25 pM of each forward and reverse primer (Sigma Aldrich), and 2 µL of DNA template in a total volume of 50 µL. Each PCR run was accompanied by a negative control reaction containing ddH_2_O in place of DNA template. The PCR reactions were subjected to the following temperature cycling conditions; (1) 95°C for 5 min., (2) 95°C for 1 min., (3) temperatures between 52°C to 63°C (primer dependant) for 1 min. and (4) 72°C for 30 sec. Steps 2 to 4 were repeated 39 times, followed by a final extension step (5) of 72°C for 5 min. All PCR products were subjected to gel electrophoresis on 2% agarose gels stained with GelRed and visualised under UV light.

# Restriction fragment length polymorphism (RFLP) analysis

A PCR-RFLP assay targeting the Leishmaniinae ITS1 DNA was employed to further characterise the novel trypanosomatid (refer to the full manuscript for details). The PCR reaction mix was prepared as above using DNA extracted from cultured promastigotes as template and an annealing temperature of 58°C. As controls for comparison, this reaction was carried out on genomic DNA from *Leptomonas* *seymouri*, *Leishmania turanica*, *Leishmania major* and *Wallacemonas* *collosoma* (previously *Leptomonas collosoma*). The assay was also performed on genomic DNA from *Leishmania donovani* provided by the Department of Microbiology at St Vincent’s Hospital, Sydney. All PCR products were digested using the *Hae* *III* restriction enzyme (Sigma Aldrich) according to the manufacturer’s instructions. The restriction fragments were subjected to agarose gel electrophoresis on a 3% gel stained with GelRed and visualised under UV light.

# Direct sequencing of PCR amplicons

The PCR products were excised from agarose gels using a sterile scalpel blade. Amplicons were extracted from gel slices using a QIAquick Gel Extraction Kit (QIAGEN) according to the manufacturer’s instructions, though with one modification; an elution volume of 33 μL of ddH_2_O. The concentration of eluates was determined using a Nanodrop ND-1000 Spectrophotometer and adjusted for sequencing using ddH_2_O in accordance with the recommendations of the sequencing service provider. All sequencing was performed by the service provider Macrogen (South Korea) on an ABI 3730XL capillary sequencer, at least twice in each direction (forward and reverse). The .AB1 sequence files generated were analysed using either of the freely available software, 4Peaks (nucleobytes) for Mac OS, or Chromas (Technelysium) for PC. Ambiguous, low quality bases were manually trimmed from the ends of sequences which were then assembled using CAP3. Finally, BLASTN and BLASTX searches were carried out to confirm the identity of the sequences. As a control for this sequencing workflow, sequences were generated for *L. seymouri* using the primers designed in this study, for comparison to *L. seymouri* sequences already available in GenBank.

# Sequencing of cloned PCR amplicons

Sequences generated from PCR amplicons of *gGAPDH* and *RPOIIL* displayed several ‘dual-peaks’, where two bases were superimposed at the same base position along the sequence. Furthermore, the multi-copy ITS1 DNA sequences of trypanosomatids sometimes differ between copies, so clean chromatograms cannot be obtained by direct sequencing of ITS1 amplicons. Cloning of these amplicons was performed to investigate the nature of these dual peaks and to determine the sequence of several ITS1 DNA copies. Following extraction from gel slices, these amplicons were cloned using a TOPO TA cloning kit for sequencing, which included One shot Top 10 chemically competent *Escherichia coli* cells and the pCR4-TOPO TA Cloning Vector (Thermo Fisher Scientific). Cloning reactions were prepared according to the manufacturer’s instructions. Transformants were struck onto pre-warmed LB agar plates containing 50 μg/mL kanamycin (the selective agent), and incubated at 37°C overnight. The following day, 10 colonies per plate (30 for ITS1 amplicons) were individually picked with a sterile loop and used to inoculate 5 mL of LB broth containing 50 μg/mL kanamycin. Broth cultures were incubated again overnight at 37°C. Cloning reactions were accompanied by a positive control supplied with the kit. To confirm that the correct amplicon was cloned, 1 µL of each LB broth culture was boiled in 10 µL of ddH_2_O for one minute. Two µL of this solution was then subjected to the same PCR protocol that generated the original amplicon. The resulting PCR products were subjected to agarose gel electrophoresis (2% agarose) as described above. Plasmid DNA was only extracted from PCR-positive LB broth cultures using an ISOLATE II Plasmid Mini Kit (Bioline), in accordance with the manufacturer’s instructions. Sequencing was carried out directly from the purified plasmid, twice in the forward and reverse directions, by the service provider Macrogen. Sequencing was performed using the universal T3 and T7 primers which possess priming sites flanking the amplicon insertion site.
