# Supplementary material for: Isolation of Novel Trypanosomatid, Zelonia australiensis sp. nov. (Kinetoplastida: Trypanosomatidae) Provides Support for a Gondwanan Origin of Dixenous Parasitism in the Leishmaniinae
Source: PLoS Negl Trop Dis. 2017 Jan 12;11(1):e0005215. doi: 10.1371/journal.pntd.0005215 (PMC5230760; doi:10.1371/journal.pntd.0005215)
Supplement: S1 Table — This table lists the GenBank accession numbers for all nucleotide sequences used to construct phylogenetic trees in this study. (DOCX) [file pntd.0005215.s005.docx]

# Supplementary Table 1. Sequences used in phylogenetic analyses.

| **Species** | **GenBank accession number for each sequence** | | | |
| --- | --- | --- | --- | --- |
|  | ***18S rRNA*** | ***gGAPDH*** | ***RPOIIL*** | ***HSP70*** |
| *Crithidia acanthocephali* | AUXI01000956 | - | AUXI01000685 | - |
| *Crithidia brachyflagelli* | JF717840 | JF717835 | - | - |
| *Crithidia brevicula* | KJ443353 | KJ443343 | - | - |
| *Crithidia fasciculata* | Y00055 | AF047493 | AODS02000476 | AODS02000483 |
| *Crithidia permixta* | EU079127 | EU076607 | - | - |
| *Endotrypanum colombiensis* | KF302750 | - | KM820662 | - |
| *Endotrypanum equatorensis* | KF041802 | - | DQ383655 | - |
| *Endotrypanum monterogeii* | JQ863389 | AOFS01000215 | AOFS01000668 | AOFS01000512 |
| *Herpetomonas costoris* | JQ359728 | JQ359738 | - | - |
| *Herpetomonas muscarum* | JQ359731 | DQ092548 | AUXJ01002445 | AUXJ01009699 |
| *Herpetomonas nabiculae* | JN624300 | KF054088 | - | - |
| *Herpetomonas pessoai* | JQ359718 | JQ359732 | - | - |
| *Leishmania amazonensis* | JX030083 | APNT01001811 | AF009154 | HF586354 |
| *Leishmania arabica* | ATBH01001173 | - | ATBH01001028 | - |
| *Leishmania braziliensis* | JX030135 | XM_001566870 | AF009155 | GU071180 |
| *Leishmania donovani* | X07773 | XM_003862962 | AF009157 | JX021431 |
| *Leishmania enriettii* | ATAF02000704 | - | ATAF02000511 | - |
| *Leishmania gerbilli* | ATBK01001059 | - | ATBK01000855 | - |
| *Leishmania hoogstraali* | KF041810 | - | AF009162 | - |
| *Leishmania infantum* | GQ332359 | - | FR796463 | - |
| *Leishmania lainsoni* | KF041805 | - | FJ817494 | - |
| *Leishmania major* | X53915 | XM_001684852 | AF009163 | HF586344 |
| *Leishmania mexicana* | GQ332360 | XM_003877392 | AF009164 | HF586401 |
| *Leishmania naiffi* | KF041807 | - | FJ817496 | - |
| *Leishmania panamensis* | JN003595 | XM_010703012 | AF009165 | XM_010702330 |
| *Leishmania macropodum* | AY495829 | - | HM775497 | - |
| *Leishmania* martiniquensis | ATAD02000183 | - | ATAD02000494 | - |
| *Leishmania* sp. 'siamensis' | KJ467218 | - | KM820664 | - |
| *Leishmania tarentolae* | M84225 | DQ092549 | AF009166 | AY423868 |
| *Leishmania tropica* | GQ332363 | ATAT01000168 | AF009167 | Y08020 |
| *Leishmania turanica* | ATBU01001538 | - | ATBU01000353 | - |
| *Leptomonas acus* | DQ910923 | DQ910926 | - | - |
| *Leptomonas podlipaevi* | DQ383649 | DQ019001 | - | - |
| *Leptomonas pyrrhocoris* | JN036653 | JN036651 | XM_015808464 | XM_015798028 |
| *Leptomonas seymouri* | AF153040 | AF047495 | AF338253 | KY273519* |
| *Lotmaria passim* | AHIJ01002555 | - | AHIJ01002112 | - |
| *Novymonas esmeraldas* | KT944309 | KT944300 | - | - |
| *Phytomonas serpens* | AF016320 | EU084893 | AIHY01001087 | AIHY01014119 |
| *Porcisia hertigi* | KF302747 | - | AF009161 | - |
| *Zelonia australiensis* | KY273498* | KY273496* | KY273492* | KY273497* |
| *Zelonia costaricensis* | DQ383648 | DQ383650 | DQ383651 | - |

*Sequences generated as part of the present study.
